# HPMC Hydrogel Formation Mechanisms Unveiled by the Evaluation of the Activation Energy

**DOI:** 10.3390/polym14030635

**Published:** 2022-02-07

**Authors:** Saray Perez-Robles, Claudia Carotenuto, Mario Minale

**Affiliations:** Department of Engineering, University of Campania “Luigi Vanvitelli”, Real Casa dell’Annunziata, Via Roma 29, 81031 Aversa, Italy; saray.perezrobles@unicampania.it (S.P.-R.); mario.minale@unicampania.it (M.M.)

**Keywords:** rheology, activation energy, hydroxypropyl methylcellulose, inverse thermogelation, phase separation, viscoelasticity

## Abstract

Aqueous solutions of hydroxypropyl methylcellulose (HPMC) show inverse thermoreversible gelation, i.e., they respond to small temperature variations exhibiting sol–gel transition during heating, and reversibly gel–sol transition during cooling. According to the pertinent literature on HPMC aqueous systems, at room temperature, the loss modulus (G”) is higher than the storage modulus (G’). During the heating ramp, the viscoelastic response follows a peculiar path: initially, G” and G’ smoothly decrease, then drop to a minimum and finally increase. Eventually, G’ overcomes G”, indicating the gel formation. A recent explanation of this behaviour considers a two-step mechanism: first, phase separation occurs, then fibrils form from a polymer-rich phase and entangle, leading to a three-dimensional network. Based on this, our research focuses on the rheological analysis of the different steps of the sol–gel transition of an HPMC aqueous solution. We perform different viscoelastic tests: thermal ramps, time sweeps, and frequency sweeps at selected characteristic temperatures. We couple classical analysis of the SAOS experiments with an innovative approach based on the evaluation of the activation energy (*Ea*), made possible by the instrument intrinsic temperature oscillations around the target value. Results show that *Ea* can be a valid tool that contributes to further clarifying the peculiar microstructural evolution occurring in this kind of thermoreversible gel.

## 1. Introduction

Biopolymers derived from cellulose are widely used in the food [1], cosmetic [2,3], biomedical [4,5], pharmaceutical [6,7,8], construction [9,10], and oil and gas [11,12] industries due to their tightening up and shape memory properties, film formation, and barrier skills, along with boil-out and bursting avoidance at high temperatures.

Cellulose, the most abundant organic polymer available on Earth, is a polysaccharide formed by a linear chain of β (1→4) linked D-anhydroglucose units (AGU) [13]. In its raw form, it is highly insoluble in both water and nonpolar organic solvents due to hydroxyl groups spread along the backbone that form strong intra- and intermolecular hydrogen bonds [14]. Still, the partial substitution of hydroxyl groups with small substituents, such as, e.g., methyl [15,16,17], ethyl [18,19], hydroxypropyl [20,21], and carboxyl [22,23], turns cellulose into water-soluble derivatives because the substituents hinder the formation of the intra- and intermolecular hydrogen bonds, thus allowing the hydration of the AGU units. The substitution of the hydroxyl groups makes the polymer amphiphilic and might provide the property of undergoing thermoreversible gelation on temperature variations that would allow expansion of its use [24]. Thermoreversible gelation of cellulose derivatives aqueous solutions must be understood as sol–gel transition, to form a hydrogel when the temperature is increased and can be reversed by simple cooling [25].

We focus on hydroxypropyl methylcellulose (HPMC), which contains both methoxy and hydroxypropyl groups as substituents of the hydroxyl groups. The average number of methyl groups per AGU is known as the degree of substitution (DS), and the average hydroxypropyl molar content is called molar substitution (MS). Typically, commercially available samples of HPMC have DS between 1.3 and 2.1 and lower MS ranging from 0.1 to 1.0 [26].

HPMC sol–gel transition has been studied by many authors with rheological measurements under small amplitude oscillatory flow [27,28,29,30,31,32,33,34,35]: initially, the dynamic moduli during a heating ramp slowly decrease up to a first critical temperature T_A_ where they drop, reaching a minimum at a second critical temperature T_B1_ for G’ and T_B2_ for G”. T_B2_ may be also few degrees larger than T_B1_. After the minimum, the moduli increase with the temperature and G’ eventually overcomes G”, indicating the gel formation.

**Figure 1:**
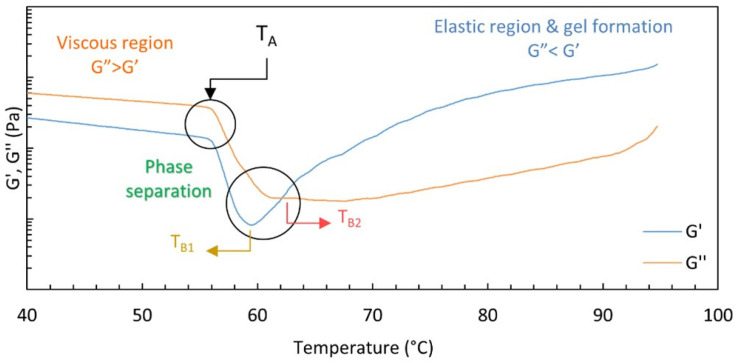
G’ and G” vs. temperature: typical behaviour of HPMC* inverse thermogelation. The temperatures corresponding to the beginning of the phase separation (T_A_) and the minima of the moduli (T_B1_ and T_B2_) are shown. (*Mn: ~86 kDa, DS: 1.48, MS: 0.2, from Merck).

In 2009, Bodvik et al. [31] found that T_A_ is independent of polymer concentration and that the T_B1_ feature is more evident for samples with higher DS. They were also pioneering in observing polymer aggregation in the form of “globular objects” owing to the “bulkiness” of the hydroxypropyl groups through Cryo-TEM measurements. Later, Fairclough et al. [32] proposed that liquid–liquid phase separation, after the drop in G’, took place through “spinodal decomposition” forming a bicontinuous structure with polymer-rich and polymer-poor regions before gelation. Meanwhile, Shahin et al. [33] speculated that a permanent network with large pores is formed by crosslinking of few quantities of HPMC fibrils, and then a transient network forms with most of the polymer after T_A_ filling the pores, explaining the moduli minimum as a collapse in the transient network. Most recently, Lodge et al. [34] interpreted these results by invoking a two-step mechanism: first, when T > T_A_, liquid–liquid phase separation occurs, leading to polymer-rich droplets immersed in a polymer-depleted matrix with a consequent important reduction of the system moduli, then for T > T_B_ (within the range T_B1_, T_B2_) fibrils form and associate, resulting in a three-dimensional network with the resulting increase of the moduli and their crossover. The fibrils are imagined as originating from the polymer-rich droplet phase. An example of HPMC thermogelation behaviour is shown in Figure 1, where the critical temperatures are also indicated.

Many questions remain open regarding the kinetic and thermodynamic behaviour of HPMC. This work contributes to understanding the mechanisms of inverse thermogelation by proposing a new investigation method based on the estimation of the activation energy (*Ea*) of the system in the different phases of the process. To this end, oscillatory thermal ramps are coupled with frequency spectra and time sweep tests at selected critical temperatures.

## 2. Materials and Methods

HPMC was purchased from Merck KGaA (Darmstadt, Germany). According to the manufacturer, the HPMC characteristics follow: Mn ~90 kDa, DS = 1.1 (21.0 wt%), MS = 0.11 (5.0 wt%), and the viscosity of the 2.0 wt% HPMC aqueous solution is 21.1 Pa·s at 20 °C.

The HPMC aqueous solution was prepared following a protocol proposed in the literature [34]. The polymer in its powder form was dried overnight at 60 °C. Subsequently, the desired amount of dried polymer was dispersed in one-third of distilled hot water (80 °C); vigorous stirring was imposed for 15 min until no lumps were visible; immediately after, the necessary quantity of cool water was added. To assure complete hydration of the polymers, the mixture was stirred for at least 4 h in an ice bath. The solution was stored in a refrigerator at 4 °C and used after a week of preparation when the entrapped air bubbles had disappeared. In this work, a single concentration of 3.0 wt% was studied.

**Figure 2:**
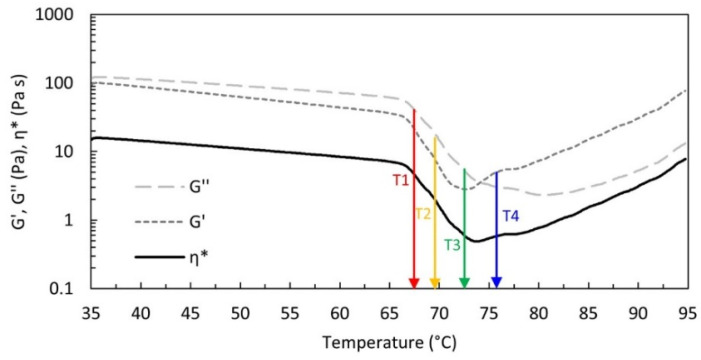
G’, G”, and ƞ* vs. temperature measured with a strain of 10%, and a frequency of 10 rad/s during a 1 °C/min heating ramp. The coloured arrows indicate the selected temperatures (67.5, 69.5, 72.5, 75.5 °C) chosen for the time and frequency sweep tests.

All measurements were performed with a strain-controlled rheometer ARES-G2 (TA Instruments) equipped with a ± 0.1 °C precision Peltier cell. A Couette geometry with a 27.6 mm DIN bob and 30 mm diameter cup was used. To avoid water evaporation, the sample was sealed with a thin layer of light silicone oil (0.1 Pa·s, more than two orders of magnitude smaller than the viscosity of the HPMC aqueous solution at 20 °C) together with a solvent trap. To individuate the small amplitude oscillatory shear (SAOS) conditions during the entire process, a preliminary linearity check was performed at different temperatures. The thermogelation, i.e., the phase separation and the subsequent gelation, was followed by SAOS, with strain amplitude of 10% and angular frequency (ω) of 10 rad/s, during a heating ramp of 1 °C/min from 30 to 90 °C. To better investigate the microstructural evolution of the system, time sweep tests of at least 20 min and frequency sweep tests were run at selected critical temperatures: the first temperature, T1, stands for the beginning of phase separation, typically evident due to a remarkable change of slope in the viscoelastic moduli and in the complex viscosity (ƞ*); the second, T2, matches the middle of the phase separation branch; the third, T3, corresponds to the minimum of G’, i.e., when the gelation starts to overcome the phase separation, and the fourth temperature, T4, occurs soon after the crossover between G’ and G’’, in the region where the gel is better assessed (see Figure 2).

## 3. Results and Discussion

### 3.1. Time Sweep Tests

**Figure 3:**
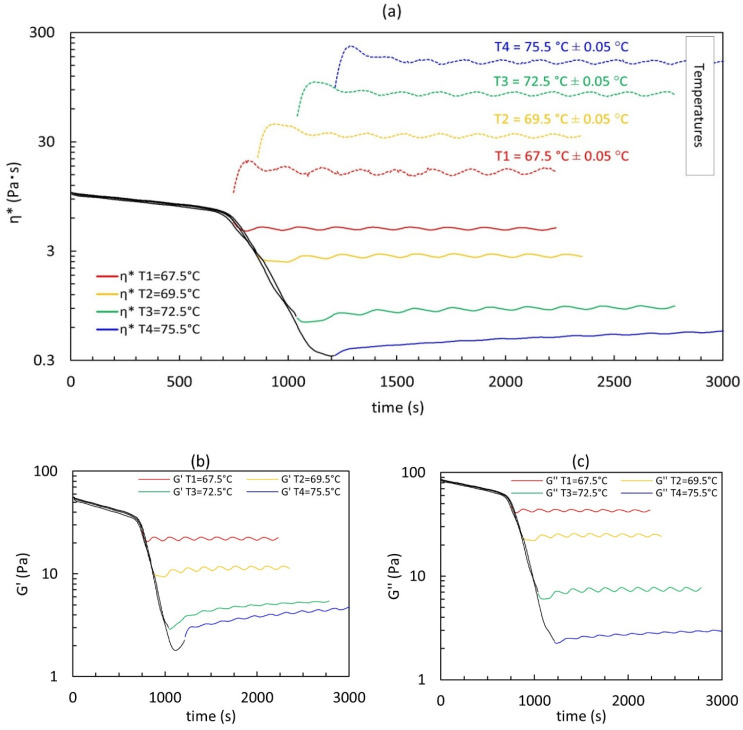
Time sweep test results in terms of (**a**) complex viscosity; (**b**) elastic modulus; (**c**) viscous modulus at the four selected temperatures. Data from thermal ramps run to reach the selected temperatures are also plotted in black. Graph (**a**) also shows measured temperatures as a function of time during time sweep tests. Strain amplitude is 10% and frequency is 10 rad/s.

Time sweep tests at the four selected temperatures were run after a thermal ramp at 1 °C/min, from 30 °C to the selected temperature. After each time sweep test, the system was cooled back to 30 °C. At 30 °C, before running any new thermal ramp, the system was sheared at $\dot{\gamma }$ =10 s^−1^ for 600 s to erase any flow history. The viscosity measured at 30 °C was always the same (62.34 ± 2.74 Pa·s), confirming the full reversibility of HPMC thermogelation. In Figure 3, we show the complex viscosity measured during each thermal ramp followed by the time sweep experiment vs. time. The temperatures vs. time during the time sweep experiments are also shown as a reference.

We first notice the very good data reproducibility in the sol phase region, for T < T_A_, and in the phase separation one, for T_A_ < T < T_B_. We also notice that the temperature during the time sweep experiments passes through a very small overshoot before reaching the target value around which oscillates with a period of about 170 s and an amplitude of 0.05 °C, well within the specifications of the Peltier cell used. Interestingly, the complex viscosity also oscillates after an undershoot following both the temperature oscillations and overshoot, respectively. The same happens to both G’ (Figure 3b) and G” (Figure 3c), and we will analyse this later in Section 3.3: “Activation energy (*Ea*) evaluation”.

Some of the data of Figure 3, in the time sweep region, show an average increase with time, superimposed to the oscillations. This slight increase with time is more evident at higher temperatures (T3 and T4) and suggests that the microstructure evolves at a constant temperature. To better quantify this, the mean dimensionless slope of the evolution with time of η*, G’, and G” is evaluated and reported in Table 1. The slopes are calculated with a linear regression, Equation (1), by interpolating the time sweep data not affected by the initial transient consequent to the temperature overshoot shown in Figure 3a. The first-order coefficient, *b*, is then normalized with the mean value of the data used for the interpolation:

*y* = *a* + *b*
*t*. (1)

**Figure 4:**
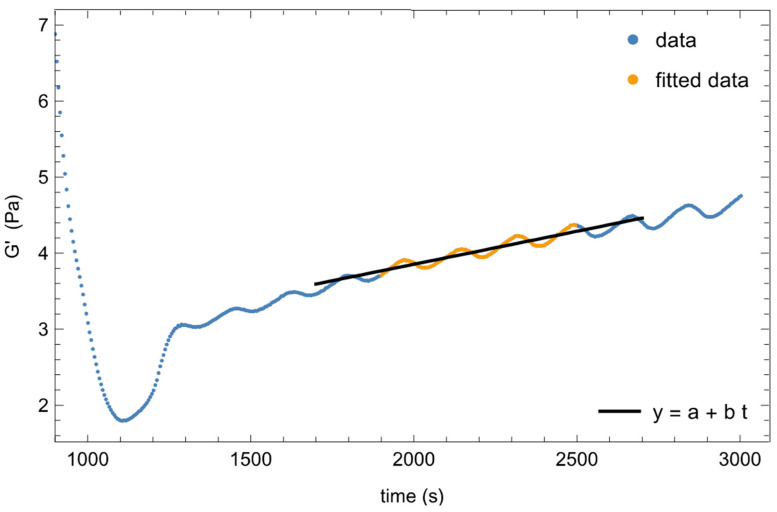
Example of the linear interpolation with Equation (1) to calculate the normalized slope. T = 75.5 °C. Data used for the interpolation are shown in orange; Equation (1) is the black straight line.

An example of the linear fit is shown in Figure 4 with the data used for the interpolation shown in orange.

From the data of Table 1, it is clear that a dimensionless slope of *O*(10^−5^) is within the experimental error and can thus be considered as zero. Nondimensional slopes smaller than *O*(10^−4^) are then reported in grey in Table 1 to highlight that these values can be actually considered nil. From the analysis of the slopes, it is clear that when the system is in the phase separation region (T1 and T2), the microstructure does not evolve in time at a constant temperature; conversely, when the system is in the gel-formation region (T4), the microstructure evolves in time at a constant temperature and G’ increases faster than G” indicating that the elasticity builds more rapidly than the viscous stiffening. At an intermediate temperature, T3, which corresponds to the transition zone from the phase separation to the gel region, we see that G’ increases with time while G” remains unchanged, the complex viscosity also increases very slowly at a rate of the order of the experimental error. This suggests that the gel is at the beginning of its formation; at this early stage the material is mainly viscous, such that the increase of the number of physical bonds with time induces an elasticity increase, but it is not enough to stiffen the system.

### 3.2. Frequency Sweep Test

**Figure 5:**
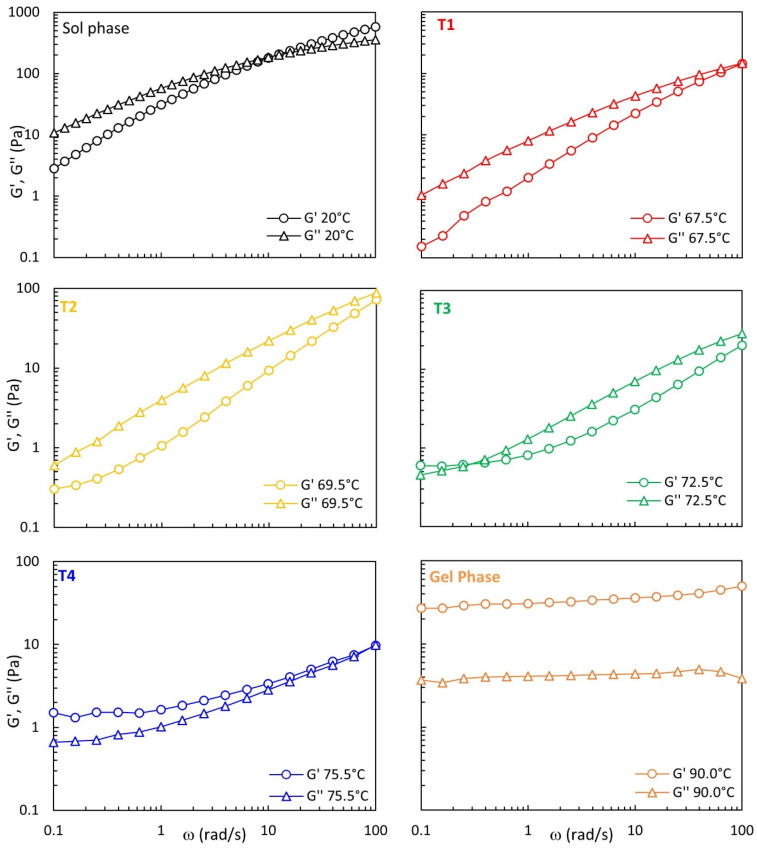
G’ and G’’ during the frequency sweep tests at different temperatures with a constant imposed strain of 10%.

The gel build-up can be investigated with the frequency sweep tests carried out at the four selected temperatures. The elastic and viscous moduli vs. the angular frequency are shown in Figure 5. As a reference, the frequency sweep results at 20 °C (in the sol phase) and at 90 °C (when the gel is formed) are also shown. In the former case, the classical spectra of a viscoelastic solution are obtained; in particular, the moduli crossover is at about 10 rad/s and the terminal zone is approached for angular frequencies smaller than 0.1 rad/s. Similarly, in the latter case, the spectra typical of a gel are obtained, in fact, the viscoelastic moduli are essentially parallel and independent of the frequency, with G’ much larger than G”, indicating a well-entangled network.

At T1, a sol behaviour is still dominant, and the viscoelastic weakening induced by the temperature increase is evident both from the shift of the crossover frequency towards higher values and from the reduction of G’ and G”. However, the terminal zone at low frequencies of G’ is not clearly visible, either because of the instrument sensibility or because some elasticity is starting to build-up. At T2, the liquid–liquid phase separation, according to the literature [32,34], is occurring and we notice that the moduli increase with the increase of temperature. A clear elasticity is now evidenced by the values of G’ at small frequencies, which depart from the typical terminal zone of a sol phase. This may be due to either the viscoelasticity of the polymer-rich phase or the build-up of a gel network. At T3, the elasticity at small frequencies is more evident and G’ overcomes G” indeed. By increasing the frequency G’ crosses G” and more classical sol behaviour is again observed with a second crossover that can be guessed at higher frequencies. At these two temperatures, T2 and T3, data indicate that the system is mainly a viscoelastic fluid with a classic moduli crossover at the high frequency, but also with an elastic solid-like fingerprint, with a long characteristic relaxation time. Finally, at T4, we have the clue of the gel formation as G’ is everywhere larger than G” and the moduli are both practically horizontal at small frequencies. However, we should emphasise that at T3 and T4 the microstructure evolves in time at a constant temperature, in particular, as shown in Figure 3 and Table 1, at T3 G’ increases with time, while G” remains mainly constant, and at T4 G’ increases with time more rapidly than G”.

Let us now consider that to take most of the spectra points immediately after the temperature has reached the set point, the frequency sweep experiments are run by decreasing the frequency, so the data at low frequencies are those taken at the end of the experiment when an increase in moduli may have taken place. Thus, the moduli behaviour shown at small frequencies may be affected by the microstructure change in time at a constant temperature.

The spectra at T2 and T3 are compatible both with a viscoelastic fluid formed by a more elastic polymer-rich phase immersed in a viscoelastic polymer-poor phase, and with a viscoelastic fluid where a 3D network is forming. To try to discern between these two hypotheses, we took advantage of the intrinsic temperature oscillations during the time sweep tests (upper part of Figure 3a) that allowed us to estimate the *Ea* of the system during the different phases of the thermogelation process.

### 3.3. Activation Energy (Ea) Evaluation

First, we evaluated the activation energy (*Ea*) of the system in the sol phase, for T < T_A_, using data recorded during the thermal ramp at 1 °C/min (Figure 2). It can be calculated according to an Arrhenius-like equation (see Equation (2)) from the variation of the complex viscosity with the temperature. Analogously, we can estimate the magnitude of the temperature dependence of the viscoelastic moduli by evaluating *Ea* of G’ and G” by fitting Equation (2) through the data of the moduli vs. temperature:

(2) $$p=k\;e^{Ea/RT},$$

where *p* can be ƞ*, G’, or G”; *k* is a pre-exponential factor; *Ea* is the activation energy; *R* is the gas constant; and *T* is the absolute temperature (K).

From the data of Figure 3, we obtained the positive *Ea* value reported in Table 2, i.e., the larger the temperature, the smaller the complex viscosity. The activation energies calculated from G’ and G” are also positive, and that calculated from G’ is larger than that calculated from G”. This implies that in the sol phase the elastic modulus is more sensitive to the temperature variation; it weakens more rapidly than the viscous modulus.

Similarly, we can estimate the activation energies from the variation of the complex viscosity and of the moduli with the temperature in the phase separation branch of Figure 2, i.e., for T_A_ < T < T_B_, using the Arrhenius-like equation (Equation (2)). We obtain positive values, reported in Table 2, that are one order of magnitude larger than those of the sol phase. Additionally, in this case, *Ea* calculated from G’ is larger than that calculated from G”. These are apparent activation energies, however, since in the phase separation branch by varying the temperature we simultaneously probe two mechanisms: the typical variation of viscoelastic parameters with temperature, similarly to what is observed in the sol phase, and the change of the microstructure related to the formation of polymer-rich and polymer-depleted regions; the more phase-separated, the weaker the system. Let us emphasise that we also run the thermal ramp at a slower velocity and it superimposes to the one here discussed at 1 °C/min. This suggests that during the phase separation branch, the microstructure is at its equilibrium, at any temperature.

**Figure 6:**
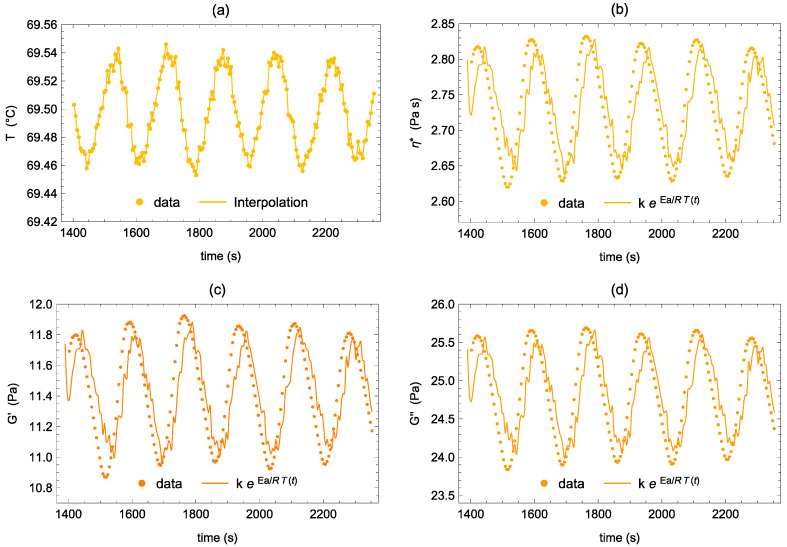
Example of *Ea* estimation using the time sweep data at T2 (69.5 °C). (**a**) Third-order polynomial interpolation of the temperature oscillating signal around the set-up value vs. time; (**b**–**d**) Data and their fitting through Equation (2) where T(t) is the interpolation of (**a**): (**b**) complex viscosity, (**c**) elastic modulus, and (**d**) viscous modulus.

To decouple the two mechanisms, we took advantage of the intrinsic temperature oscillation around its set-up value to estimate *Ea* from the time sweep experiments at the four selected temperatures. To this end, we first interpolated the temperature oscillatory signal vs. time by using third-order polynomial curves between successive data points. Figure 6a shows, as an example, the interpolation of temperature data at T2 = 69.5 °C. Then, with the T(t) function obtained, we fitted Equation (2) through ƞ*, G’, and G” data vs. time (Figure 6b–d). In order to have an estimation of *Ea* not affected by the average increase of the moduli and of the complex viscosity with time, particularly evident at T3 and T4 (see Figure 3 and Table 1), we fitted the data after having subtracted from them the values due to this increase in time, calculated with Equation (1). We fitted the same range of data used to calculate the average evolution in time of the rheological parameters (orange points in Figure 4). As shown in Figure 6, the fitting is more than satisfactory in all cases. The values of the activation energies calculated at the four characteristic temperatures are listed in Table 2. Let us emphasise that the oscillation period of the temperature is about 170 s, while the time sweep test is run at 10 rad/s, and each experimental point is collected in one oscillatory cycle of 0.628 s, during which the variation of the temperature can be considered negligible. Moreover, as the microstructure was in its equilibrium state during the thermal ramp at 1 °C/min, we can assume that it is also in its equilibrium state at each temperature during the oscillations. Furthermore, the amplitude of the temperature oscillation is 0.05 °C, and it is too small to induce a measurable change of the rheological properties with the temperature if the microstructure does not change. Indeed, this small temperature variation in the sol region (T < T_A_) does not cause a measurable data fluctuation. Consequently, with these time sweep tests, we will actually probe the sole response of the system to a change of microstructure induced by the change of temperature.

At temperature T1, we measure positive activation energies for the three rheological parameters, and they are very comparable to the respective values calculated from the whole phase separation branch (see Table 2). At T2, well within the phase separation region, once again the activation energies are all positives and about double those previously estimated from the full branch. This seems to suggest that a more phase-separated system is more sensitive to temperature variations. At temperature T3, in a region approaching the beginning of the gel formation, the responses of ƞ*, G’, and G” differ. Compared to the value at T2, the *Ea* calculated from the complex viscosity remains almost unvaried, the *Ea* calculated from G’ significantly decreases, remaining positive, while the *Ea* calculated from G” measurably increases. We might explain this behaviour by imagining that the polymer-rich phase is sufficiently concentrated that a minimum increase of its concentration induced by the increase of temperature is accompanied by a significant increase of elasticity that partially compensates for the reduction of the modulus due to the progress of the phase separation. Similarly, the viscous response, dominated by the polymer-poor phase, is more sensitive to the concentration variation induced by the temperature changes. Finally, at temperature T4 in the gel phase, we detect a change of sign of the *Ea* calculated from G’ and ƞ*, but not from G”. This implies that by increasing the temperature, G’ and ƞ* increase while G” still decreases. This is quite in agreement with the behaviour shown in Figure 2, where the inverse thermogelation is depicted. We are thus probing the gel response to the temperature where the higher the temperature, the more elastic the gel. At T4, the viscous modulus keeps reducing with the temperature because the physical bond density is still too low to limit the viscous dissipation after an imposed deformation.

To summarize the main results obtained from this analysis, we first highlight the change of sign of the *Ea* of G’ and η* passing from T3 to T4, which is in perfect agreement with a transition from a phase separation region, where the behaviour of the system is still liquid-like, to a gel region, where the system is solid-like. Since the gelation investigated is an inverse thermogelation, the negative value of the *Ea* at T4 is perfectly sound. Data calculated at T3 suggest that the increase of elasticity recorded during the frequency sweep test at this temperature can be reasonably related to the phase separation into a polymer-rich elastic phase and a polymer-poor one, more than to the onset of the 3D gel network. In fact, in the latter case, the *Ea* calculated from G’ should have been negative. The increase of *Ea* passing from the data at T1 to those at T2 can be explained considering that at T1 the phase separation involves the system marginally and thus the sol behaviour still affects the system response significantly. More generally, data suggest that the more phase-separated the system, the more sensitive to the temperature variation. This is confirmed by the *Ea* calculated from G” at T3.

## 4. Conclusions

The inverse thermoreversible gelation of an aqueous solution of HPMC at 3.0 wt% was rheologically investigated with SAOS experiments. First, a classical thermal ramp at 1 °C/min, at a fixed angular frequency (10 rad/s), was performed and three regions were individuated: a sol phase, followed by a phase separation region, and eventually by a gelphase. Subsequently, at selected characteristic temperatures (T1, T2, T3, and T4 of Figure 2), both time sweep experiments and frequency sweep tests were executed. Time sweep tests showed that in the phase separation region the system very rapidly reaches an equilibrium state that does not evolve in time, while in the gelation region, it evolves in time at a constant temperature. This suggests that the dynamics of the phase separation, or the pseudo phase separation, is very fast while that of the gelation is much slower. The frequency spectra allowed for highlighting the build-up of system elasticity with increase of temperature. The spectra were, however, compatible with two possible physical interpretations: (A) the system separates into a polymer-rich viscoelastic phase and a polymer-poor phase, the higher the temperature the more phase-separated the system; (B) the gel starts to form since the first critical temperature and the network characteristic dimension, i.e., the average distance between two physical bonds, decreases with the temperature, and the bonds density increases with it. Hypothesis A agrees with the most recent theories [34].

We introduced a new approach to discern between the two hypotheses based on the estimation of the activation energy (*Ea*) of the system in the different thermodynamic states. This was done by exploiting an instrumental intrinsic very small oscillation of the temperature (±0.05 °C) around the set-up value during the time sweep experiments. Results clearly show that when the system passes from the sol state to the phase separation one, the *Ea* calculated from ƞ*, G’, and G’’ undergo a steep increase, changing more than one order of magnitude. Moreover, when the system starts to form the gel, only the *Ea* from G’ changes sign, marking the onset and growth of a 3D network, such that the higher the temperature the more elastic the system.

The evaluation of the *Ea* from the temperature oscillations during the time sweep experiments allowed us to support hypothesis A regarding the mechanism of gel formation. Indeed, while approaching the temperature of the gel onset, the measured increase of elasticity coupled with the positive *Ea* calculated from G’ can be nicely explained by invoking the phase separation with the formation of an elastic polymer-rich phase. We can then interpret the frequency sweep data within this scenario, and we can suggest that the phase separation might start from a very coarse and soft microstructure that becomes less coarse and more elastic as the temperature increases. Indeed, the elasticity, highlighted by G’ data, shows up first at small frequencies and then extends to higher frequencies, as the temperature increases; the lower the frequency where the elasticity emerges, the longer the relaxation time and the coarser the microstructure. For the sake of clarity, we must emphasise that we cannot be conclusive on this point since we showed that the microstructure evolves in time, at a constant temperature, significantly at T4, in the gel region, but also at T3, in the phase separation region. Thus, our frequency sweep data are also affected by this unavoidable microstructural change.

## Figures and Tables

**Table 1:** Dimensionless slope of time sweep data shown in Figure 3. The dimensionless slopes are calculated from η*, G’, and G plots.

Slope	T1 67.5 (°C)	T2 69.5 (°C)	T3 72.5 (°C)	T4 75.5 (°C)
from ƞ*	−1.73·10^−5^	−4.39·10^−6^	4.33·10^−5^	1.79·10^−4^
from G’	−1.20·10^−5^	2.34·10^−5^	1.18·10^−4^	2.16·10^−4^
from G”	−1.87·10^−5^	−1.03·10^−5^	7.78·10^−6^	1.02·10^−4^

**Table 2:** Activation energy, *Ea/R* (K) in the different phases of the thermogelation process calculated from η*, G’, and G”. The standard error of the parameter estimate is also reported. R is the universal gas constant.

	*Ea*/*R* (K) Sol Phase	*Ea*/*R* (K) Phase Separation	*Ea*/*R* (K) Time Sweep
Calculated from η*	2.85·10^3^ ± 26	4.92·10^4^ ± 5.8·10^2^	T1: 4.27·10^4^ ± 2.4·10^3^ T2: 8.84·10^4^ ± 4.9·10^3^ T3: 8.10·10^4^ ± 4.6·10^3^ T4: −2.32·10^4^ ± 1.7·10^3^
Calculated from G’	3.48·10^3^ ± 29	5.73·10^4^ ± 5.9·10^2^	T1: 5.29·10^4^ ± 2.9·10^3^ T2: 1.01·10^5^ ± 5.5·10^3^ T3: 3.69·10^4^ ± 2.2·10^3^ T4: −5.12·10^4^ ± 4.0·10^3^
Calculated from G”	2.62·10^3^ ± 26	4.77·10^4^ ± 6.1·10^2^	T1: 4.00·10^4^ ± 2.3·10^3^ T2: 8.57·10^4^ ± 4.7·10^3^ T3: 1.02·10^5^ ± 4.6·10^3^ T4: 3.50·10^4^ ± 3.5·10^3^

## Data Availability

The data presented in this study are available on request from the corresponding author.
